# Genetic and transcriptomic dissection of host defense to Goss's bacterial wilt and leaf blight of maize

**DOI:** 10.1093/g3journal/jkad197

**Published:** 2023-08-31

**Authors:** Yangfan Hao, Ying Hu, Jennifer Jaqueth, Jinguang Lin, Cheng He, Guifang Lin, Mingxia Zhao, Jie Ren, Tej Man Tamang, Sunghun Park, Alison E Robertson, Frank F White, Junjie Fu, Bailin Li, Sanzhen Liu

**Affiliations:** Department of Plant Pathology, Kansas State University, Manhattan, KS 66506, USA; Department of Plant Pathology, Kansas State University, Manhattan, KS 66506, USA; Corteva Agriscience, Johnston, IA 50131, USA; Department of Plant Pathology, Kansas State University, Manhattan, KS 66506, USA; Department of Plant Pathology, Kansas State University, Manhattan, KS 66506, USA; Department of Plant Pathology, Kansas State University, Manhattan, KS 66506, USA; Department of Plant Pathology, Kansas State University, Manhattan, KS 66506, USA; Department of Plant Pathology, Kansas State University, Manhattan, KS 66506, USA; Department of Plant Pathology, Kansas State University, Manhattan, KS 66506, USA; Department of Horticulture and Natural Resources, Kansas State University, Manhattan, KS 66506, USA; Department of Plant Pathology, Entomology and Microbiology, Iowa State University, Ames, IA 50010, USA; Department of Plant Pathology, University of Florida, Gainesville, FL 32611, USA; Chinese Academy of Agricultural Sciences, Institute of Crop Science, Beijing 100081, China; Corteva Agriscience, Johnston, IA 50131, USA; Department of Plant Pathology, Kansas State University, Manhattan, KS 66506, USA

**Keywords:** maize disease, bacterium, Goss's wilt, disease resistance, XP-GWAS, QTL, Plant Genetics and Genomics

## Abstract

Goss's wilt, caused by the Gram-positive actinobacterium *Clavibacter nebraskensis*, is an important bacterial disease of maize. The molecular and genetic mechanisms of resistance to the bacterium, or, in general, Gram-positive bacteria causing plant diseases, remain poorly understood. Here, we examined the genetic basis of Goss's wilt through differential gene expression, standard genome-wide association mapping (GWAS), extreme phenotype (XP) GWAS using highly resistant (R) and highly susceptible (S) lines, and quantitative trait locus (QTL) mapping using 3 bi-parental populations, identifying 11 disease association loci. Three loci were validated using near-isogenic lines or recombinant inbred lines. Our analysis indicates that Goss's wilt resistance is highly complex and major resistance genes are not commonly present. RNA sequencing of samples separately pooled from R and S lines with or without bacterial inoculation was performed, enabling identification of common and differential gene responses in R and S lines. Based on expression, in both R and S lines, the photosynthesis pathway was silenced upon infection, while stress-responsive pathways and phytohormone pathways, namely, abscisic acid, auxin, ethylene, jasmonate, and gibberellin, were markedly activated. In addition, 65 genes showed differential responses (up- or down-regulated) to infection in R and S lines. Combining genetic mapping and transcriptional data, individual candidate genes conferring Goss's wilt resistance were identified. Collectively, aspects of the genetic architecture of Goss's wilt resistance were revealed, providing foundational data for mechanistic studies.

## Teaser

Using whole genome and transcriptomic sequencing of extremely resistant and susceptible maize lines to the Gram-positive bacterial disease, Goss’s wilt, along with traditional genome-wide association mapping, quantitative trait locus analysis, and comparison of near-isogenic lines, Hao et al. identified association loci and the genes commonly and differentially responding to the bacterium in resistant and susceptible maize lines. Integration of omics’ data led to the findings of multiple well-evidenced candidate genes that are responsible for host resistance.

## Introduction

Goss's bacterial wilt and leaf blight (GW), caused by the Gram-positive actinobacterium *Clavibacter nebraskensis* (Cn) (formerly *Clavibacter michiganensis* subsp. *nebraskensis*), is an important maize disease (Schuster 1972, 1975; Vidaver 1981; Li *et al.* 2018). Infection by Cn primarily happens through physical damages to leaves caused by rain, hail, or wind-blown sand. GW symptoms are characterized by large, elongated tan lesions with wavy margins that originate from wounds on or the tips of leaves. The lesions subsequently turn yellow and then become necrotic (Claflin 1999; Jackson *et al.* 2007). The disease is widespread in the Upper Midwest of the United States and Canada (Langemeier *et al.* 2017; Webster *et al.* 2020).

GW development is largely influenced by host resistance to Cn (Langemeier *et al.* 2017). Infection of susceptible varieties can result in 40–60% yield loss, and this is greatly reduced if Cn-resistant maize hybrids are planted (Robertson 2012). A few studies have been conducted on the genetic basis underlying host resistance to GW. A quantitative trait locus (QTL) mapping study using the population derived from 3 nested-associated mapping (NAM) recombinant inbred lines (RIL) families identified 11 QTLs on chromosomes 1, 2, 3, 4, 5, 9, and 10 (Singh *et al.* 2016). Another bi-parental QTL mapping study using B73×Mo17 derived populations found QTLs on chromosomes 1, 2, 7, 8, 10 (Cooper *et al.* 2018). A genome-wide association study (GWAS) using historical Minnesota maize inbred lines identified 9 loci associated with disease resistance to GW (Schaefer and Bernardo 2013), while a recent GWAS study using a 550 diverse inbred lines and 450 RIL found 4 associated loci on chromosomes 1, 2, 5, each of which explained 1–5% of the phenotypic variation (Singh *et al.* 2019). No GWAS peaks were identified using the Goodman maize diversity panel (Cooper *et al.* 2019). All these studies used the disease phenotype from adult plants and showed resistance to GW is a complex and polygenic trait. An alternative approach used quantified disease symptoms on the seedlings of 615 maize lines and employed the analysis of extreme phenotype copy number variation (XP-CNV) to identify disease defense-associated CNV, including an association with the locus *rp1* on the short arm of chromosome 10 (Hu *et al.* 2018). However, no specific resistance genes to actinobacteria have been reported.

The host defense to Cn is expected to share similar mechanisms to tomato upon infection with *Clavibacter michiganensis* (Cm). No secretion systems equivalent to the type III or IV effector secretion systems (T3SS or T4SS) have been identified in Cn or Cm. Consequently, recognition of pathogen-associated molecular patterns (PAMPs) is thought to play an important role in plant defense to Cn and Cm. Proteases have been shown to elicit a nonhost resistance in the case of Cm and *Clavibacter sepedonicus* (Cs) (Nissinen *et al.* 2009; Lu *et al.* 2015; Verma and Teper 2022). Bacterial cold shock proteins, cell wall components, exopolysaccharides, extracellular cell wall degrading enzymes secreted by bacteria, and even degraded fragments from the host cell wall may function as PAMPs to elicit plant defense (Balaji *et al.* 2008). A proteomic study revealed differences in protein expression between high and low virulent strains of Cn when grown in maize xylem sap (Soliman *et al.* 2021). Recently, *Walls Are Thin1* (*WAT1*) in tomato was shown to be a susceptibility gene to Cm. Inactivation of *WAT1* enhanced tomato resistance to diverse Cm strains, likely through reducing auxin and ethylene (ET) content in plants, as well as suppressing the production of virulence factors in bacteria (Koseoglou *et al.* 2023).

Transcriptional studies have provided expression profiles of host responses to the *Clavibater* bacterium. Comparison of transcriptomic responses to Cn infection between an R line and an S line reported many genes that respond to infection by Cn are involved in regulation of metabolism (Singh *et al.* 2019). A transcriptomic analysis that compared infection of tomato by the closely related species Cm, with mock inoculation revealed many basal defense-related genes, which are involved in activity of reactive oxygen species, protein turnover, and hormone biosynthesis such as ET biosynthesis, were induced upon infection with Cm. Several putative cell-surface receptors showing differential expression in response to Cm infection were identified (Balaji *et al.* 2008).

Extensive genetic materials and genomic information regarding maize and Cn have become available, improving our ability to study both the genetics of host resistance and bacterial virulence (Romay *et al.* 2013; Bukowski *et al.* 2018; Hu *et al.* 2018). Here, we examined the genetic basis for GW resistance by mapping disease resistance loci through QTL mapping, standard GWAS, and extreme phenotype (XP) association analyses that relied on bulked pools of individual maize lines that displayed R or S phenotypes. Combining genetic, genomic, and transcriptomic analyses aid in identification of genomic loci and candidate genes relevant to Goss's wilt resistance.

## Materials and methods

### Plant materials

In total, 615 diverse maize inbred accessions or lines were used for multiple analyses. Seeds of maize lines were ordered from North Central Regional Plant Introduction Station (NCRPIS). All these lines have been phenotyped in 2016 to quantify the GW disease symptom in our previous study, identifying 37 highly resistant (R) lines and 44 highly susceptible (S) lines to GW (Hu *et al.* 2018). In this study, additional 3 R and 7 S lines were identified. In total, we identified 40 R lines and 51 S lines from the whole set of 615 lines (Supplementary Fig. 1). The 615 lines included 418 inbred lines in two association mapping populations, Pop410 and Pop269 (Supplementary Fig. 1, Supplementary Tables 1 and 2). The Pop410 population consists of 410 lines that were previously genotyped with genotyping-by-sequencing (GBS) (Supplementary Table 1) (Romay *et al.* 2013). Another population Pop269 consists of 253 accessions from the maize 282 association panel (Bukowski *et al.* 2018) and 16 R or S lines that are not from the maize 282 association panel (Supplementary Table 2). All lines of Pop269 were genotyped through whole genome sequencing (WGS). Pop269 shares 254 lines with Pop410. From Pop269, 33 R and 31 S lines were used for the extreme phenotype genome-wide association analysis (Supplementary Fig. 1, Supplementary Table 3).

Besides, 3 RILs were utilized to localize the linkage region and compare with the association mapping results. RILs from 2 NAM families [B73×M37W (BM) and B73×Hp301 (BH)] were from NCRPIS and the Intermated B73×Mo17 (IBM) RILs were shared by Patrick Schnable at Iowa State University.

### Leaf clipping bacteria inoculation

The leaf clipping inoculation method was established previously (Hu *et al.* 2018). Briefly, the inoculum of the virulence strain CMN06-1 prepared from culture in the Nutrient Broth Yeast medium was used for the clipping inoculation at 2 cm from the tip on the third leaf of the 3-leaf maize seedlings. CMN06-1 is a strain isolated in an Iowa maize field (Hu *et al.* 2018). The bacterial inoculum in the potassium phosphate buffer (12.5 mM, pH 7.1) was prepared to the optical density of 0.55–0.60 at 600 nm. The lesion length was scored from the clipping tip to the lesion margin close to the ligule of leaves at 13 days after inoculation (DPI). A set of common lines were used as the control in different phenotyping batches. As defined in the previous study, lines with lesion length shorter than 9 cm were defined as R lines and lines with lesion length longer than 22 cm were defined as S lines (Hu *et al.* 2018).

### Phenotyping of association populations

Seeds were germinated in Metro mix 360 soil in the greenhouse at 28°C during the daytime and 21°C at night with a 16/8 h light/dark photoperiod. Pop410 (*N* = 410) inbred lines were phenotyped in 2016. Pop269 (*N* = 269) inbred lines were phenotyped in 2016 and 2018. At least 3 replicates per inbred line were performed. A linear model was employed to obtain best linear unbiased estimates of inbred lines. In the model, the raw lesion length was a response variable, and genotype of each line was an explanatory variable. Year, batch, and the interaction between year and genotype were used as covariates. In the model, genotype was treated as a fixed factor, while year, batch, and the interaction between year and genotype were treated as random factors.

### Genotyping data of Pop410

Of 615 phenotypically scored lines, genotyping data of 410 maize lines (Pop410) that were publicly available (ZeaGBSv2.7) were used for standard GWAS (Romay *et al.* 2013). Using the filtering criteria of the minor allele frequency of at least 5% and the genotyping missing rate of at least 50%, 255,197 SNPs were retained for GWAS.

### Genotyping data of Pop269

WGS data of 254 lines of Pop269 are publicly available (Bukowski *et al.* 2018). In addition, we performed WGS of 15 R and S inbred lines for which no WGS data were available. Briefly, leaf tissues were used for genomic DNA extraction with DNeasy Plant Mini Kit (Qiagen). At least 10 × coverage of paired-end 2 × 150 bp Illumina sequencing data was produced for each line. Raw reads from all lines of Pop269 were trimmed with the Trimmomatic software to clean the adaptor and low-quality sequences (Bolger *et al.* 2014). Clean paired-end reads were aligned to the B73 reference genome sequence (B73v3) with Burrows-Wheeler Aligner (BWA) (Li and Durbin 2010). Reads uniquely mapped to the reference genome were retained. Read alignments were input to GATK for variant calling (McKenna *et al.* 2010). SNPs (*N* = 14,294,315) with the minor allele frequency (MAF) higher than 5% and the genotyping missing data rate less than 30% were kept for standard GWAS.

### Standard GWAS

Standard GWAS was separately carried out using data from Pop410 and Pop269.

GWAS analysis was performed with a mixed linear model (Yu *et al.* 2006) with the TASSEL 5.0 program (Bradbury *et al.* 2007). In TASSEL 5.0, the top three principal components and a kinship matrix were constructed to correct for population structure and family relatedness. Significant associations between markers and lesion lengths were declared if the *P*-value of a marker was smaller than 1/*N*, where *N* is the independent marker number estimated by PLINK software (Purcell *et al.* 2007). Neighboring significant SNPs within the associated region with the linkage disequilibrium (LD) of at least 0.1 were considered to belong to the same GW associated locus.

### Extreme phenotype genome-wide association study

WGS of 33 R and 31 S lines were used for variant calling through GATK (version 3.3-0-g37228af) (McKenna *et al.* 2010). Only variant sites that were biallelic and supported by at least 150 reads from all 64 lines were retained. The variants discovered by GATK were further filtered. For each variant, a logistic regression was fitted using the “glm” function in R with the family “binomial”. The response variable in the model is read counts of 2 alleles, which were assumed to follow a binomial distribution. The phenotypic group is the variable, which has 2 levels, R and S. The likelihood ratio test was employed to test the null hypothesis that there is no association between allele frequency and the phenotypic group (R or S). At the same time, an odds ratio and a *P*-value of each marker was obtained. To control the type I error (α) at the level of 5%, the significant association was declared when a *P*-value was less than the Bonferroni-adjusted significance threshold (3.8 $\times 10^{-9}$).

### QTL analysis

QTL analysis was conducted using 3 bi-parental populations, including two NAM families (179 RILs from BM and 194 RILs from BH) and 95 RILs from the IBM RILs (Lee *et al.* 2002; Yu *et al.* 2008). Three individual plants per RIL were phenotyped. In the same greenhouse environment, disease phenotyping of these RIL populations was performed in 2017. The lesion length of each RIL was evaluated by the linear mixed model similar to the one used in the association panels. Note that both B73 and Mo17 exhibited certain levels of resistance to Cn based on seedling lesion lengths. The major reason to select the IBM RIL population was to examine whether different resistant loci were employed in the two lines. SNP markers obtained from the Panzea website (http://www.panzea.org) were used to construct the linkage maps for both NAM families using the R package ASMap (Taylor and Butler 2017). A previously constructed IBM genetic map derived from RNA-seq data was used for IBM RIL QTL mapping (Lin *et al.* 2013). Three genetic maps with 6,000, 6,000, and 4,892 SNPs were used for QTL analyses of BH RILs, BM RILs, and IBM RILs, respectively. QTL analysis was performed using the R package R/qtl (Broman *et al.* 2003). We employed the interval mapping to perform the genome scan of each population by using the “scanone” function with the parameter of “method = em”. The markers with LOD values higher than the top 5% of the maximum LODs from 1,000 permutation tests were declared as significant QTLs. The QTL interval and the percentage of phenotypic variance explained by a QTL were determined by using “lodint” and “fitqtl” functions, respectively.

### Identification of the colocalization from multiple mapping results

When comparing standard GWAS and Extreme phenotype GWAS (XP-GWAS), 2 significant mapping loci were considered to be colocalized if they were within 500 kb. To examine whether GW disease-associated loci identified by GWAS or XP-GWAS were supported by QTLs from bi-parental populations, each QTL position was extended with 15 Mb at both sides from the lead SNP to delineate a 30 Mb interval consisting of approximately one-tenth to one-fifth of a chromosome. If an associated locus from GWAS or XP-GWAS was in an extended QTL interval, the locus was considered to be supported by the QTL. The loci with multiple lines of mapping evidence were further merged if adjacent loci were within 1 Mb.

### Validation of GWAS peaks

We used the NAM RIL population to validate the candidate GW associated loci. For each locus, RILs with heterozygosity in a 20 Mb interval around the locus were selected for heterogeneous inbred family analysis (Tuinstra *et al.* 1997). The RIL family (Z016E0164, a NAM RIL) from a cross of the B73 and M37W family and the RIL family (Z021E0134, a NAM RIL) from the B73 and NC358 family were selected to validate *gw1a* and *gw8a*, respectively. Based on the genotyping data of markers in the interval, homozygous lines (near-isogenic lines, NILs) were selected to quantify lesion lengths upon Cn inoculation. For *gw3b*, 25 NAM RILs with the B73 genotype at *gw3b* and 25 NAM RILs with the NC358 genotype from the B73×NC358 RIL family were used for the phenotypic comparison.

### RNA sequencing and data processing

RNA-seq was performed to compare gene expression between pooled R and pooled S lines. Five biological replicates were conducted. In each replicate, the third leaf of a 3-leaf of an R or S seedling was treated with CMN06-1 using the clipping inoculation method as previously described (Hu *et al.* 2018). The mock treatment with the PBS buffer was used as the control. At 24 h postinoculation, leaf tissues, 2 cm from the clipping regions, were collected from 37 R lines and 44 S lines and were pooled separately (Supplementary Table 3). After pooling tissues R lines or S lines, RNA was extracted using the RNeasy Plant Mini Kit (Qiagen) with the treatment of DNase I. RNA quality was assessed using Bioanalyzer (Agilent). Sequencing libraries were prepared in the Integrated Genomics Facility at Kansas State University and sequenced in the Genome Sequencing Facility at University of Kansas Medical Center for sequencing. Paired-end 2 × 100 bp Illumina reads were generated at a HiSeq2000 sequencer.

Low-quality sequences and adaptors were trimmed with Trimmomatic (Bolger *et al.* 2014). Remaining reads were mapped to the B73 reference genome (B73v3) (Schnable *et al.* 2009) with the aligner STAR (2.7.9a) (Dobin *et al.* 2013). To test the null hypothesis that no difference in gene expression occurs between the Cn treatment and the control mock treatment, a negative binomial generalized linear model implemented in DESeq2 was used (Love *et al.* 2014). A false discovery rate (FDR) approach was employed to account for multiple statistical tests (Benjamini and Hochberg 1995). The 5% FDR was set to define the statistical significance. The significant genes with at least 0.6 of the absolute value of log2-fold change between two groups were deemed as the differential expression genes (DEGs). Gene ontology (GO) enrichment analysis was performed using GOSeq (Young *et al.* 2012).

### Differential responses to Cn in R lines and S lines

To test the null hypothesis that no differential response in gene expression between R and S pools upon inoculation with Cn, a model including the resistance type (R and S), the treatment (Cn inoculation and mock treatment), and the interaction between the resistance type and the treatment was built and implemented with DESeq2 (Love *et al.* 2014). The FDR of 0.05 was set to define significant genes whose expression was influenced by the interaction between the resistance type and the treatment.

## Results

### GW resistance and genetic architecture of populations for association mapping

**Fig. 1.**
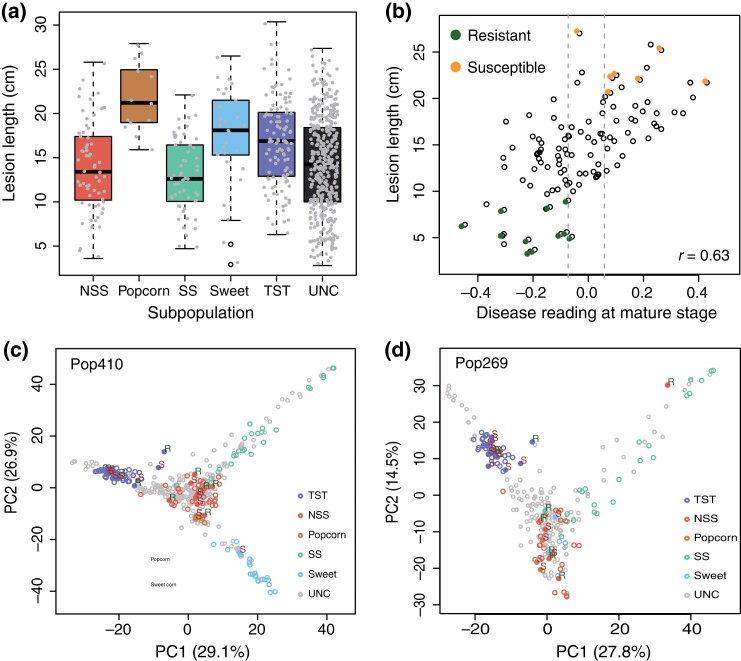
Lesion length and PCA of maize lines phenotyped for reaction to Cn infection. a) Lesion lengths of maize inbred lines in each subpopulation. b) Correlation of resistance to the Cn pathogen between performance at the seedling stage and the mature stage; Resistant and susceptible lines we identified based on seedling-stage lesion lengths are highlighted in color. The correlation is significant at the type I error (α) of 0.05. c, d) PCA of Pop269 and Pop410, open dots represent maize lines that were not classified to either resistant or susceptible lines. Solid dots stand for resistant or susceptible inbred lines. R, resistant; S, susceptible; NSS, nonstiff stalk; SS, stiff stalk; Sweet, sweet corn; TST, tropical; UNC, unclassified.

GW resistance of 615 maize inbred lines including 37 R lines and 44 S lines was quantified in lesion length at 13 DPI by clipping inoculation of the third leaf at the 3-leaf seedling stage (Hu *et al.* 2018). The lines represented 6 groups of *Zea mays* types: sweet corn, popcorn, stiff stalk (SS), nonstiff stalk (NSS), tropical lines, and an unknown group (Flint-Garcia *et al.* 2005). Lesion lengths of the inbred lines ranged from 2.8 to 30.4 cm (Supplementary Table 1, Supplementary Table 2). Among all groups, SS and NSS were more resistant than the other groups (Fig. 1a). On average, both popcorn and sweet corn lines had longer lesions than the other groups. All popcorn lines examined were susceptible (Fig. 1a). The Pearson correlation of disease phenotypes between our seedling data and mature-stage data from a previous GWAS study (Cooper *et al.* 2019) was 0.63 (P-value=0.001) and most R lines and S inbred lines identified based on seedling disease phenotyping also showed similar resistant or susceptible performance at the mature stage (Fig. 1b).

Among the 615 maize lines, genotyping of Pop410 had been previously performed with low-density markers from genome reduction GBS data (Romay *et al.* 2013). Of Pop269, all of which are from 615 phenotyped maize lines, WGS data of 254 lines are publicly available (Bukowski *et al.* 2018). We also generated WGS data, at minimum 10 × coverage per line, for the remaining 15 R or S inbred lines whose WGS data were not publicly available (Supplementary Table 4). WGS data of all these lines were combined to generate high-density SNP genotyping data of 269 lines of Pop269. Principal component analysis (PCA) of Pop410 and Pop269 populations indicated they were both collected from diverse lines (Fig. 1c and d). In this study, 269 lines of Pop269 were re-phenotyped to add additional three replicates of phenotyping data. The Pearson correlation between previously and newly phenotyped data is 0.89. All phenotyping data were combined to determine the BLUE disease phenotypes in lesion length for inbred lines in the populations of Pop410 and Pop269 (Supplementary Table 1, Supplementary Table 2). The heritability levels of Pop410 and Pop269 were 0.67 and 0.82, respectively.

### Identification of GW defense-associated loci with multiple association strategies

**Fig. 2.**
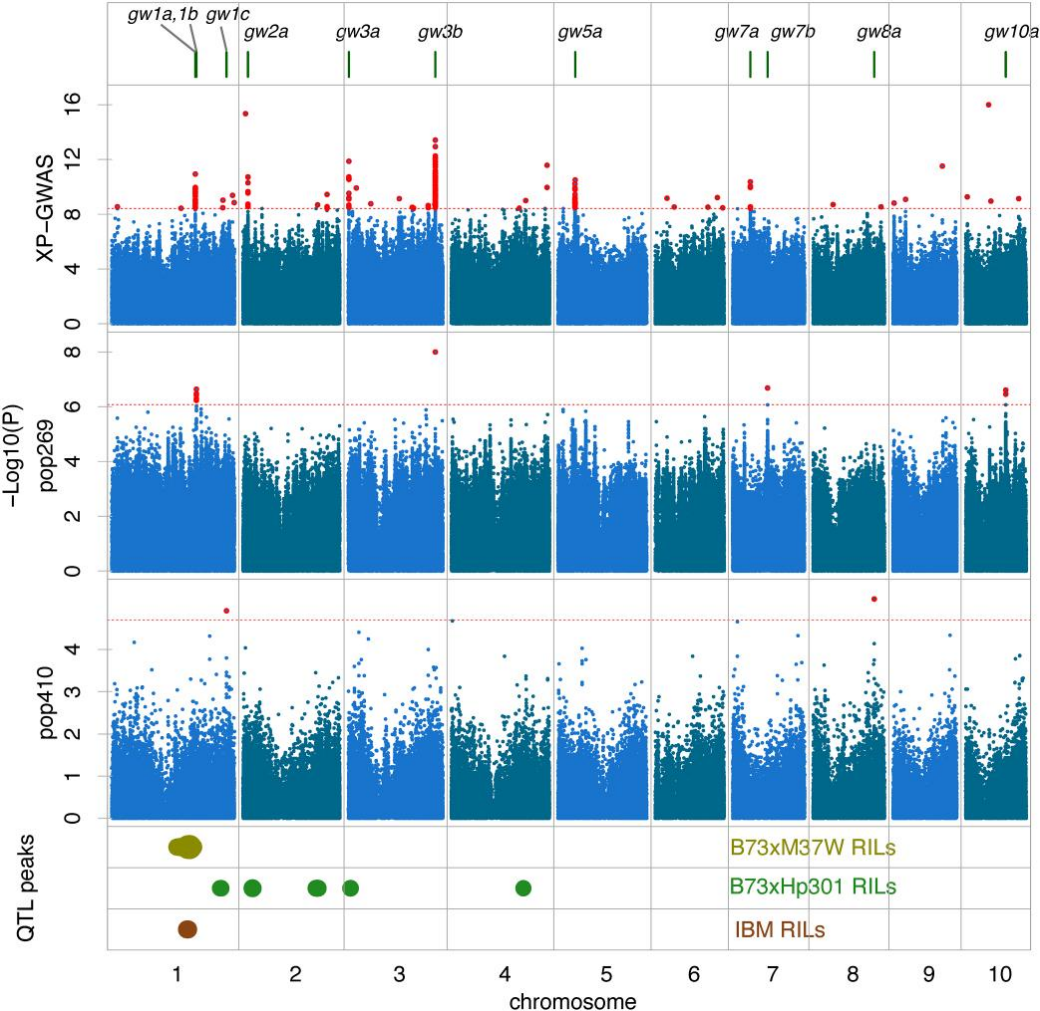
Manhattan plots of GWAS and QTL results. Three Manhattan plots were from 3 GWAS analyses. Horizontal dash lines designate the thresholds for associated SNPs. Bars on the top label 11 association loci. Dots on QTL plots stand for the QTLs from 3 bi-parental populations. Sizes of dots are positively correlated with LOD values.

GWAS using Pop410 with 255,195 SNP markers identified 2 GW disease-associated loci on chromosomes 1 and 8 (Fig. 2, Table 1, Supplementary Fig. 2a). GWAS using Pop269 with 14,294,315 high-density SNPs (Bukowski *et al.* 2018) identified 4 disease-associated loci on chromosomes 1, 3, 7, and 10 (Fig. 2, Table 1, Supplementary Fig. 2b). XP-GWAS performed on 33 R lines and 31 S lines from Pop269 identified 7 candidate loci on chromosomes 1, 2, 3, 5, and 7. In total, 11 disease-associated loci (*gw1a*, *gw1b*, *gw1c*, *gw2a*, *gw3a*, *gw3b*, *gw5a*, *gw7a*, *gw7b*, *gw8a*, and *gw10a*) were identified (Fig. 2, Table 1). Among these associated SNPs, *gw3b* was supported by both the Pop269 GWAS and XP-GWAS. SNP chr3_213572727 (213,572,727 bp on chromosome 3) from the Pop269 GWAS and chr3_213577529 from XP-GWAS are closely linked and occur in a region with a high LD (*r*^2^ = 0.86). The mapping interval of each disease-associated locus was defined by the LD block where the lead marker, the marker with the lowest *P*-value locally, was located, i.e. markers within the block had the LD of at least 0.1 with the lead marker (Table 1).

**Table 1.** Eleven GW association loci.

Locus	Chr	Start*^a^*	End*^a^*	Lead SNP position*^a^*	Allele	MAF*^b^*	*P*-value (analysis)	Supporting QTL/locus	Supporting population
*gw1a^c^*	1	205,268,605	205,334,589	205,283,374	G/C	0.4	1.10 × 10^−10^ (XP-GWAS)	*qBM1*/*qIBM1a*	XP-GWAS, BM, IBM
*gw1b^c^*	1	207,950,325	208,041,833	207,952,164	A/G	0.2	6.92 × 10^−7^ (Pop269)	*qBM1*/*qIBM1a*	Pop269, BM, IBM
*gw1c*	1	281,730,865	281,777,134	281,768,673	A/G	0.33	1.19 × 10^−5^ (Pop410)	NA	Pop410
*gw2a^c^*	2	12,360,852	13,005,895	12,395,863	A/T	0.06	1.88 × 10^−11^ (XP-GWAS)	*qBH2a*	XP-GWAS, BH
*gw3a*	3	547,406	1,521,853	1,518,227	C/T	0.06	1.33 × 10^−12^ (XP-GWAS)	NA	XP-GWAS
*gw3b^c^*	3	213,113,390	213,625,729	213,572,727	C/A	0.47	3.82 × 10^−8^ (Pop269)	XP-GWAS (*P* = 3.8 × 10^−14^)	Pop269, XP-GWAS
*gw5a*	5	40,950,440	42,279,823	42,194,906	C/T	0.25	3.13 × 10^−11^ (XP-GWAS)	NA	XP-GWAS
*gw7a*	7	42,805,009	43,439,617	43,345,774	G/A	0.26	4.22 × 10^−11^ (XP-GWAS)	NA	XP-GWAS
*gw7b*	7	84,200,724	86,456,479	85,650,161	A/G	0.34	6.24 × 10^−7^ (Pop269)	NA	Pop269
*gw8a*	8	149,693,768	150,406,954	149,985,134	C/A	0.41	6.24 × 10^−6^ (Pop410)	NA	Pop410
*gw10a*	10	98,935,931	99,222,165	99,089,574	A/G	0.38	1.19 × 10^−5^ (Pop269)	NA	Pop269

^*a*^B73v3 coordinates.

^*b*^Minor allele frequency.

^*c*^Loci supported by at least 2 analyses.

QTL analysis was performed using 2 families of NAM RILs, BM, BH, and the IBM population (Lee *et al.* 2002; Yu *et al.* 2008) (phenotypic data in Supplementary Table 5). The B73 inbred line, the common parent in the 3 RIL populations, is moderately resistant to GW (lesion length = 9.4 cm, Supplementary Table 1). The NAM parents M37W and Hp301 are susceptible (lesion lengths 23.5 and 21.2 cm, respectively), while the parent Mo17 exhibits resistance (lesion length = 8 cm). One, five, and one QTLs were identified from the mapping populations of BM, BH, and IBM, respectively (Supplementary Table 6). Three disease-associated loci from GWAS were supported by QTLs: *gw1a* and *gw1b* are both in *qBM1a* and *qIBM1a*; *gw2a* is located in *qBH2a*. Among the 11 disease-associated loci, *gw1a*, *gw1b*, *gw2a*, and *gw3b* were supported by at least 2 analyses from 3 GWAS and 3 QTL analyses.

### Validation of 3 GW disease-associated loci

**Fig. 3.**
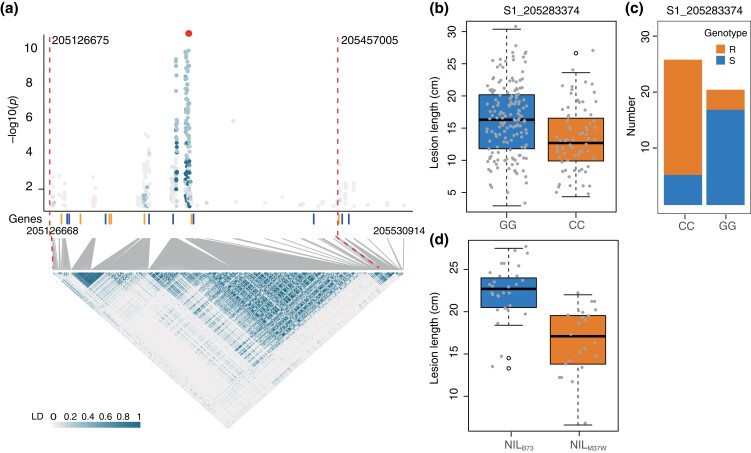
Association evidence of the *gw1a* locus with resistance to GW. a) A regional Manhattan plot and a heatmap of pairwise LDs between SNPs on the *gw1a* locus. The top dot is the lead SNP (S1_205283374) with the lowest *P*-value from GWAS. Vertical dash lines indicate the left and right flanking sites of the LD block containing SNPs having >0.1 of LD with the lead SNP. b) A boxplot of lesion lengths from 2 homologous genotypes of the lead SNP in Pop269. c) Allele numbers of 2 homologous genotypes of the lead SNP in R and S inbred lines. d) Boxplot of lesion lengths of two genotypes (NIL_B73_, NILs with B73 genotype; NIL_M37W_, NILs with M37W genotype) at *gw1a*.

The *gw1a* locus that was supported by 2 QTLs, *qBM1a* and *qIBM1a*, and XP-GWAS was examined in more detail (see Table 1). At *gw1a*, for the lead marker S1_205283374 from Pop269, the lesion length of lines containing the homozygote of G (GG) was longer than that of inbred lines containing the homozygote of C (CC) (Fig. 3a and b, *P*-value = 3.8 $\times 10^{-5}$). To test the null hypothesis that no allele difference exists in R and S sets, a $\chi^{2}$ test was performed on the allele frequency in R lines and S lines. The *P*-value calculated is 3.2 $\times 10^{-5}$, which rejected the null hypothesis and showed that genotype of most R lines (23/27) is CC, and the genotype of most S lines (19/25) is GG (Fig. 3c). In addition, lesions of NILs from a NAM RIL of BM were examined. Briefly, GBS genotyping data of BM RILs were used to find the lines with heterozygosity at the mapping location of *gw1a*. NILs with a homozygous B73 genotype (NIL_B73_) or a homozygous M37W genotype (NIL_M37W_) derived from the heterozygosity-containing lines were then identified through genotyping (Supplementary Table 7). Phenotyping of NILs showed that NIL_B73_ had longer lesions as compared with NIL_M37W_ (Fig. 3d, *P*-value = 1.0$\times 10^{-6}$), confirming the association of *gw1a* with GW resistance.

Using the same strategy, we validated the association of *gw8a* with GW resistance. *gw8a* was mapped to the interval between 149,796,829 and 150,110,318 bp on chromosome 8 (Supplementary Fig. 3). The lesion length from the homozygotes of the A allele (AA) was shorter than the homozygotes of CC at the lead marker S8_149985134 (Supplementary Fig. 3b, *P*-value = 1.6 $\times 10^{-5}$). A $\chi^{2}$ test of the numbers of 2 homozygous genotypes in the R and S sets confirmed its association with GW resistance (Supplementary Fig. 3c, *P*-value < 1 $\times 10^{-4}$). Corroboration using NILs derived from a NAM RIL of B73×NC358 showed that the B73 allele of *gw8a* conferred a longer lesion as compared with the alleles of NC358 (Supplementary Fig. 3d, α < 0.05).

**Fig. 4.**
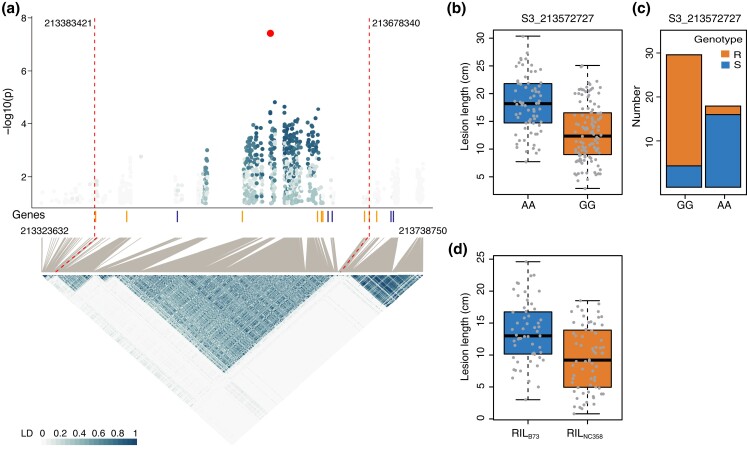
Association evidence of the *gw3b* locus. a) A regional Manhattan plot and a heatmap of pairwise LDs between SNPs on the *gw3b* locus. The top dot is the lead SNP (S3_213572727) with the lowest *P*-value from GWAS. Vertical dash lines indicate the left and right flanking sites of the LD block containing SNPs having >0.1 of LD with the lead SNP. b) A boxplot of lesion lengths from 2 homologous genotypes of the lead SNP in Pop269. c) Allele numbers of 2 homologous genotypes of the lead SNP in R and S inbred lines. d) Boxplot of lesion lengths of 2 genotypes (RIL_B73_, RILs with B73 genotype; RIL_NC358_, RILs with NC358 genotype) at *gw3b*.

The *gw3b* locus is located on an approximately 255-kb interval on chromosome 3 (Fig. 4a). In the Pop269 population, the homozygous genotype of G (GG) showed shorter lesions than the homozygous genotype of A (AA) at the lead marker S3_213572727 (Fig. 4b, *P*-value = 3.4 $\times 10^{-10}$). Of this lead marker, 26/28 R lines are with GG genotype and 17/22 S lines are with AA genotype (Fig. 4c, $\chi^{2}$ test, *P*-value = 1.0 $\times 10^{-4}$). Genotyping and phenotyping of 50 RILs from the B73×NC358 NAM family showed that the RILs with NC358 genotype at *gw3b* has shorter lesions than those with B73 genotype (Fig. 4d, *P*-value = 1.7 $\times 10^{-5}$). Our validation collectively supports the association of *gw3b* with GW resistance.

### Transcriptional responses of R and S lines to Cn infection

**Fig. 5.**
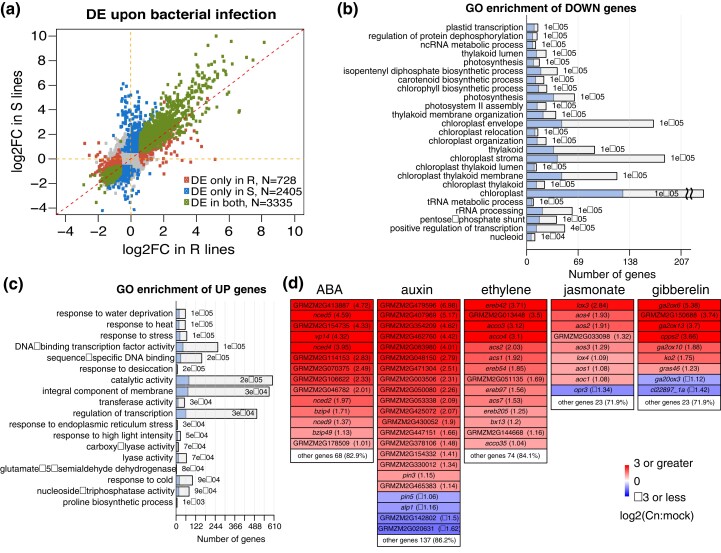
Differential expression genes in R and S pools and pathways enrichment upon Cn inoculation. a) Scatter plot of log2 of fold changes [log2FC or log2 (Cn:mock)] of gene expression upon Cn infection in resistant (R) lines and log2FC in susceptible (S) lines. DE genes are color-highlighted. b, c) Enriched GO terms in up-regulated genes (UP) b) and down-regulated genes c) in Cn-infected leaf samples as compared to control leaf samples without Cn infection. In each barplot, a colored bar stands for the number of genes in the DE gene set and the whole bar (colored and empty) stands for the total number of genes of the associated GO term. A *P*-value was labeled on each bar. d) List of significant DE genes in 5 hormone pathways. The log2FC of gene expression upon Cn infection averaged from both R and S lines are listed after each gene name. Only genes with adjusted *P*-values less than 0.05 and absolute log2FC larger than 1 are displayed.

To understand transcriptional responses upon Cn infection in both R and S lines, RNA-seq data were generated from infected leaves pooled from all R lines or pooled from all S lines (Supplementary Table 8). Leaf samples not infected with Cn (mock treatment) were used as the control. In total, 5 biological replicates were employed. Comparisons of the Cn treatment with the control resulted in 4,066 and 5,744 DEGs in R and S lines, respectively. A total of 3,335 DEGs were shared in the R and S lines and, of them, 3,334 had the same up- or down-regulations by Cn (Fig. 5, Supplementary Table 9). GO analysis of DEGs from both R and S lines found genes involved in the photosynthesis were enriched in genes down-regulated upon the Cn treatment, while stress-responsive genes were enriched in up-regulated genes (Fig. 5b and c). GO enrichment analysis also indicated that transcription factor (TF) genes were enriched in Cn-induced genes (i.e. up-regulated by Cn). TF family analysis detected TF genes in many families including EREBP, MYB, WRKY, bHLH, C2H2, NAC, and homeobox that were markedly activated by Cn (Supplementary Fig. 4).

Expression of many genes in phytohormone pathways, including abscisic acid (ABA), auxin, ET, jasmonate (JA), and gibberellin (GA) was dramatically induced by Cn infection (Fig. 5d). A high level of expression induction of the key gene of ABA biosynthesis, *vp14*, indicated that the production of ABA hormone was increased (Tan *et al.* 1997; He *et al.* 2020). Multiple small auxin-up RNA genes (e.g. GRMZM2G479596, GRMZM2G407969, and GRMZM2G354209) were strongly up-regulated by Cn, implying a marked auxin level change upon the Cn infection (Stortenbeker and Bemer 2019). All GA, JA, and ET were likely to be elevated upon Cn due to increased expression of multiple genes involved in their biosynthesis, including genes encoding ent-copalyl diphosphate synthase, ent-ekaurene oxidase (KO), and GA 2-oxidase for GA biosynthesis (Sakamoto *et al.* 2004); genes encoding allene oxide synthase and genes encoding lipoxygenases for JA biosynthesis (Wasternack and Hause 2013); and genes encoding the 1-aminocyclopropane-1-carboxylic acid (ACC) oxidase and the ACC synthase family genes for ET biosynthesis (Wang *et al.* 2002). Consistently, a recent study also indicated the plant host manipulated the levels of auxin and ET to combat the *clavibacter* infection (Koseoglou *et al.* 2023). Fewer genes involved in brassinosteroid, cytokinin, and SA were induced by Cn (Supplementary Fig. 5).

RNA-seq analysis identified 446 genes with differential responses to Cn infection between R lines and S lines (DRGs) (Supplementary Table 10). Based on fold changes of gene expression upon infection by Cn, 381 DRGs showed the same directional responses to Cn infection in both R and S lines. Moreover, in most cases (*N* = 347), S lines exhibited greater levels of bacterial responses. In contrast, 65 DRGs showed opposite expression responses to Cn. Among them, 3 are of great interest because they were up-regulated upon infection by Cn in S and down-regulated in R (Supplementary Table 10). These 3 genes are GRMZM2G102365 encoding a thioredoxin protein, GRMZM2G178074 homologous to phosphoenolpyruvate carboxylase kinase, and AC205471.4_FG007 with an unknown protein function.

### Identification of candidate GW disease-associated genes

**Fig. 6.**
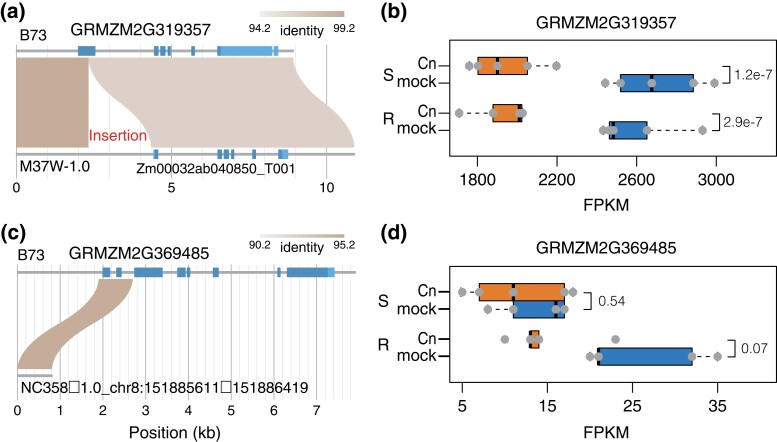
Candidates GRMZM2G319357 and GRMZM2G369485. a, c) Homologous genes of the 2 genes from B73 were separately searched in the reference genomes of M37W and NC358. For GRMZM2G319357, an ∼2 kb insertion on the first exon of the B73 allele is present on the M37W allele. For GRMZM2G369485, no intact homologs were identified in NC358. b, d) Expression responses of the genes to bacterial inoculation (Cn) and mock inoculation. Expression data were from RNA-seq in pooled samples of S and R lines. Adjusted *P*-values are labeled for comparisons between Cn and mock.

Candidate genes from 3 validated mapping intervals were prioritized, namely *gw1a*, *gw3b*, and *gw8a*. The resistance association of the *gw1a* locus was confirmed between B73 and M37W. Of 11 genes in *gw1a*, the gene prioritized is GRMZM2G319357, a Cn responsive gene in transcription (Fig. 6, Supplementary Table 11). GRMZM2G319357 encodes a low molecular weight protein-tyrosine-phosphatase, which was reported to negatively influence mouse defense to *Pseudomonas aeruginosa* infection (Yue *et al.* 2016). The QTL and NIL validation results indicated that the M37W allele of *gw1a* enhanced the resistance to GW as compared to the B73 allele. Gene structural comparison with the B73 allele showed that a large insertion is present in the first exon of the M37W allele (Fig. 6a). The presence of the insertion may disrupt the function of a negative regulator for host defense, thereby enhancing the resistance level. Based on RNA-seq of pooled R and S lines, GRMZM2G319357 was down-regulated upon Cn inoculation in both R and S pools (Fig. 6b).

The *gw3b* locus includes 8 genes (Supplementary Table 11). Two genes (GRMZM5G824843 and GRMZM5G831142) were annotated as a single gene Zm00001eb158110 in the latest genome annotation (B73v5). The gene encodes a WD40-repeat containing protein, which may be involved in plant cell wall formation and cell wall-related bacterium–host interactions (Delgado-Cerezo *et al.* 2012; Bellincampi *et al.* 2014). The association of the *gw3b* locus with the resistance level was confirmed between B73 and NC358. Comparison between B73 and NC358 found 3 presence and absence variations of large DNA fragments in an intron (Supplementary Fig. 6), 10 missense mutations, and a 3-bp insertion and deletion polymorphism at the last exon (Supplementary Table 12). Based on RNA-seq of pooled R and S lines, both GRMZM5G824843 and GRMZM5G831142 were up-regulated upon Cn inoculation in both resistant and susceptible pools.

Candidate gene GRMZM2G033515 was also selected because its ortholog in *Arabidopsis* participates in redox signaling linked to defense responses (Niazi *et al.* 2019). The gene encodes cytosolic NADP + dependent isocitrate dehydrogenase and contributes to NADPH production under oxidative stress in the cytosol. Note that GRMZM2G033515 showed differential responses to infection by Cn in R and S with a stronger response in S (Supplementary Fig. 7).

*gw8a* on chromosome 8 contains 14 genes. The gene GRMZM2G369485, a homolog with rice *glutaredoxins GRXS17*, was prioritized (Fig. 6a, Supplementary Table 11). *GRXS17* is involved in responses to the heat and drought stress (Tamang *et al.* 2021; Sprague *et al.* 2022). Based on the validation, the B73 genotype of *gw8a* corresponded to longer lesions as compared with the NC358 genotype. No intact homologous genes were identified in NC358 (Fig. 6c). The gene was down-regulated upon Cn inoculation in R pools using the 10% FDR as the threshold, while no expression change after infection by Cn was found in S pools (Fig. 6d).

## Discussion

Here, evidence for 11 GW disease association loci, each of which was supported by multiple mapping results, was obtained by various approaches. Standard GWAS controls population structure and relatedness to reduce spurious association but relies on the accuracy of phenotyping data. Disease symptom quantification was, therefore, performed using seedlings under a controlled environment, which reduced variation in our phenotypic measurements. XP-GWAS, which uses highly R and S lines, was performed as a complementary alternative to GWAS (Yang *et al.* 2015). This strategy allows a small number of phenotypic data points to be subjected to repeated tests. Consequently, XP-GWAS can be valuable for association mapping of traits that are difficult to reliably quantify. We also performed QTL mapping of 3 RIL populations that shared a common founder B73. The discrepancy between GWAS and QTL results indicates some resistance and susceptibility alleles are not segregating in these bi-parental populations. For example, the GW disease-associated locus *GW8a* detected by GWAS was not detected by bi-parental QTL analysis. Further examination confirmed that there was no variation of the GWAS lead associated SNP for *GW8a* among all 4 founders of the QTL populations.

Although all previous GW QTL or GWAS genetic mapping studies used adult plants in fields, and we used maize seedlings in the greenhouse, there are some consistencies with these GW association data. A GWAS in historical Minnesota maize inbred lines identified 9 loci associated with disease resistance to GW (Schaefer and Bernardo 2013), among which one locus overlapped with *GW1c* from this study within 1 Mb. QTL analysis using IBM RILs (N > 200) identified multiple QTLs, including a QTL on chromosome 1 overlapping with *qIBM1a* that is the only QTL detected using IBM RILs (*N* = 95) in our study (Cooper *et al.* 2018). Another QTL study using RILs (*N* = 143) of a NAM family, BH, identified 6 QTLs, among which only a QTL on chromosome 2 was close to one of the QTLs, *qBH2a*, detected using RILs (*N* = 194) from the same NAM family (Singh *et al.* 2016). The difference in QTL discoveries might be largely due to different inoculation approaches, different statistical power using distinct individuals or different number of individuals using the same population (e.g. IBM). Specifically, the *rp1* locus identified through copy number variation by comparing R and S lines (Hu *et al.* 2018) was not detected by either GWAS or QTL in our study. The resistance alleles at the *rp1* locus might represent rare alleles. In addition, a high frequency of allelic or nonallelic recombination resulting in a high level of genomic complexity and diversity could reduce the GWAS power (Hulbert 1997; Ramakrishna *et al.* 2002).

Nevertheless, our GW phenotyping data from seedlings was similar to phenotyping data using adult plants in field conditions, and thus is indicative of the common mechanisms for seedling and adult resistance to GW. Our study and previous GW genetic mapping studies consistently indicate that GW resistance is highly polygenic and major large effect genes, if exist, are rare (Cooper *et al.* 2019; Singh *et al.* 2019). We therefore hypothesized that nucleotide-binding leucine-rich repeat (NLR) gene-for-gene resistance is likely not a major mechanism in GW resistance in maize as NLR resistance typically provides high-level resistance (Kolmer 1996). However, the *rp1* locus, which contains many NLR genes, is associated with GW resistance (Hu *et al.* 2018). Identifying the GW resistance gene within this locus would be interesting and deepen insights into the genetic mechanisms of GW resistance.

Our phenotypic data indicate that 2 heterotic groups, SS and NSS, of temperate varieties generally exhibit stronger resistance than the other groups of maize we tested. Adult phenotypic data from Cooper et al. (2019) indicated NSS lines generally showed a high level of resistance to GW. In contrast, the popcorn and sweet corn contained much less genetic resources for GW resistance than those of SS and NSS lines. Popcorn was reported to be more susceptible than varieties belonging to other subpopulations at adult stages (Singh *et al.* 2019). Given GW is endemic in the United States and Canada and causes considerable yield loss (Cooper *et al.* 2019), it is critical to introduce resistant genomic loci to susceptible varieties, particularly popcorn and sweet corn. Evaluation of impacts of resistance genes or alleles in hybrids is needed for optimization of breeding strategies. Although cloning genes conferring GW resistance is a daunting task due to the trait being polygenic and small effects are associated with most resistance loci, gene identification will undoubtedly facilitate resistance breeding through either precise marker-assisted breeding or genome editing.

## Supplementary Material

jkad197_Supplementary_Data

## Data Availability

Genotyping data of Pop269 for GWAS have been deposited at the website of figshare (doi:10.6084/m9.figshare.23486867). Genotyping data for XP-GWAS have also been deposited at figshare (doi:10.6084/m9.figshare.23471201). WGS of R and S lines have been deposited to Sequence Read Archive (SRA) under accession PRJNA883391 (SRR22423504-SRR22423518). RNA-seq data of resistant and susceptible lines have been deposited to SRA under accession PRJNA905715 (SRR22421314-SRR22421333). Related scripts are available at GitHub (https://github.com/PlantG3/GWmapping). The GitHub repository also contains three genetic maps used in the QTL analysis. Supplemental material is available at G3 online.
