# Supplementary material for: Genetic and transcriptomic dissection of host defense to Goss's bacterial wilt and leaf blight of maize
Source: G3 (Bethesda). 2023 Aug 31;13(11):jkad197. doi: 10.1093/g3journal/jkad197 (PMC10627284; doi:10.1093/g3journal/jkad197)
Supplement: jkad197_Supplementary_Data [file jkad197_supplementary_data.zip › G3-2023-404466_Supplementary_Figures.docx]

Genetic and Transcriptomic Dissection of Host Defense to Goss's Bacterial Wilt and Leaf Blight of Maize

Hao et al.


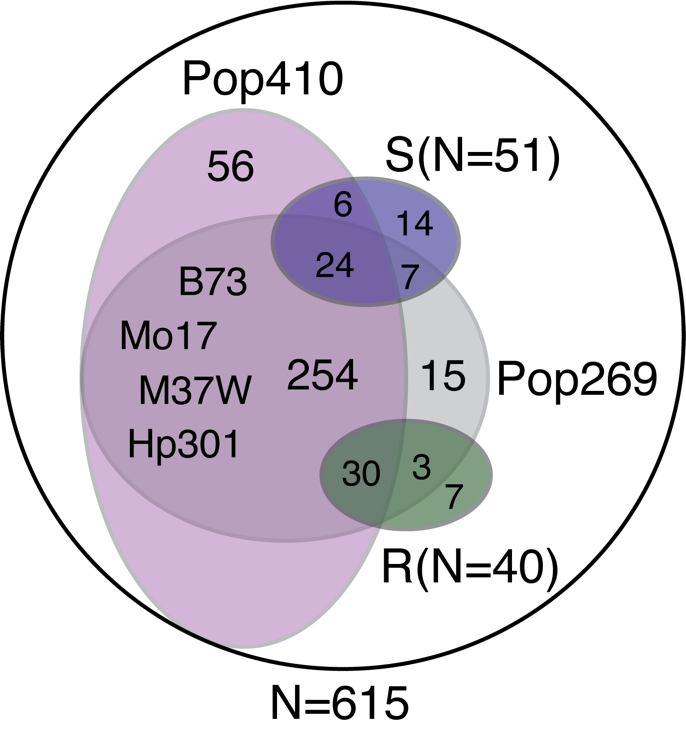


**Figure S1**. Overlaps of populations used in the study. Pop410 and Pop269 consist of 410 and 269 lines, respectively. Pop410 was selected due to the availability of genotyping data. Pop269 includes 247 lines from the maize 282 association panel and 22 R or S lines that are not in maize 282 association panel. Whole genome sequencing data of all lines in Pop269 are either publicly available or were generated in this study, providing high-density genetic markers for GWAS. Both populations are from 615 collected maize inbred lines and share 254 lines. In total, 40 R and 51 S lines were identified from the 615 lines. Among R/S lines, 37 R and 44 S lines have been used in our RNA-seq study and in our previous copy number variation study (Hu et al., 2018). Of all R/S lines, 33 R and 31 S are in Pop269, which were used for XP-GWAS. Four parents of our QTL populations are shared by Pop410 and Pop269.


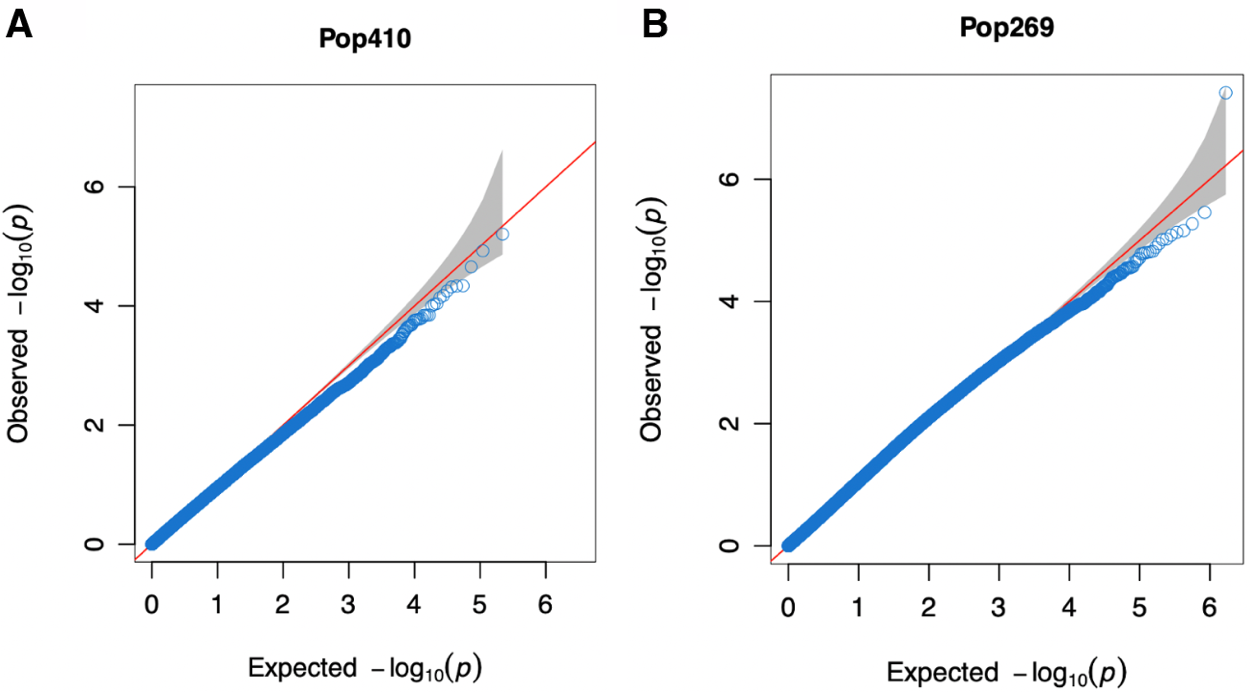


**Figure S2**. The quantile-quantile (Q-Q) plots of GWAS. (**A**) Q-Q plot of GWAS using Pop410. (**B**) Q-Q plot of GWAS using Pop269.

**Figure S3**. Association evidence at the *gw8a* locus. (**A**) A heatmap of pairwise LDs between SNPs on the *gw8a* locus. The red dot is the lead SNP S8_149985134 (B73v3) with the lowest *p*-value from GWAS. Red dash lines indicate the left and right flanking sites of the LD containing SNPs having LD (*r^2^*) greater than 0.1 with the lead SNP. (**B**) Allele numbers of two alleles of the lead SNP S8_149985134 in R and S inbred lines. (**C**) A boxplot of lesion lengths from two alleles of the lead SNP S8_149985134. (**D**) A boxplot of lesion lengths of two genotypes (NIL_B73_, NILs with B73 genotype; NIL_NC358_, NILs with NC358 genotype) at *gw8a.*


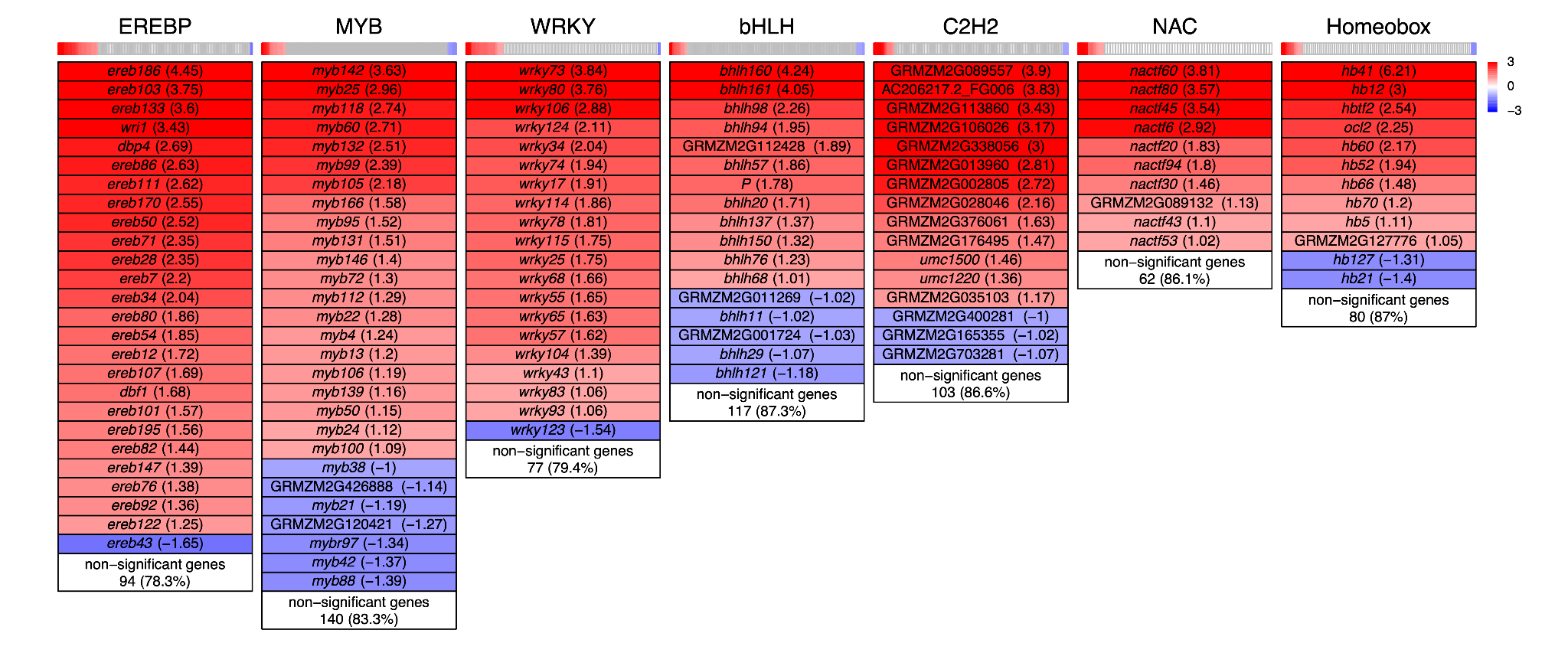


**Figure S4**. List of significant DE genes in seven families of transcription factors. The log2 Cn:mock fold change of gene expression (log2FC) averaged from both R and S lines are listed after each gene name. Only genes with adjusted p-values less than 0.05 and absolute log2FC larger than 1 are displayed.


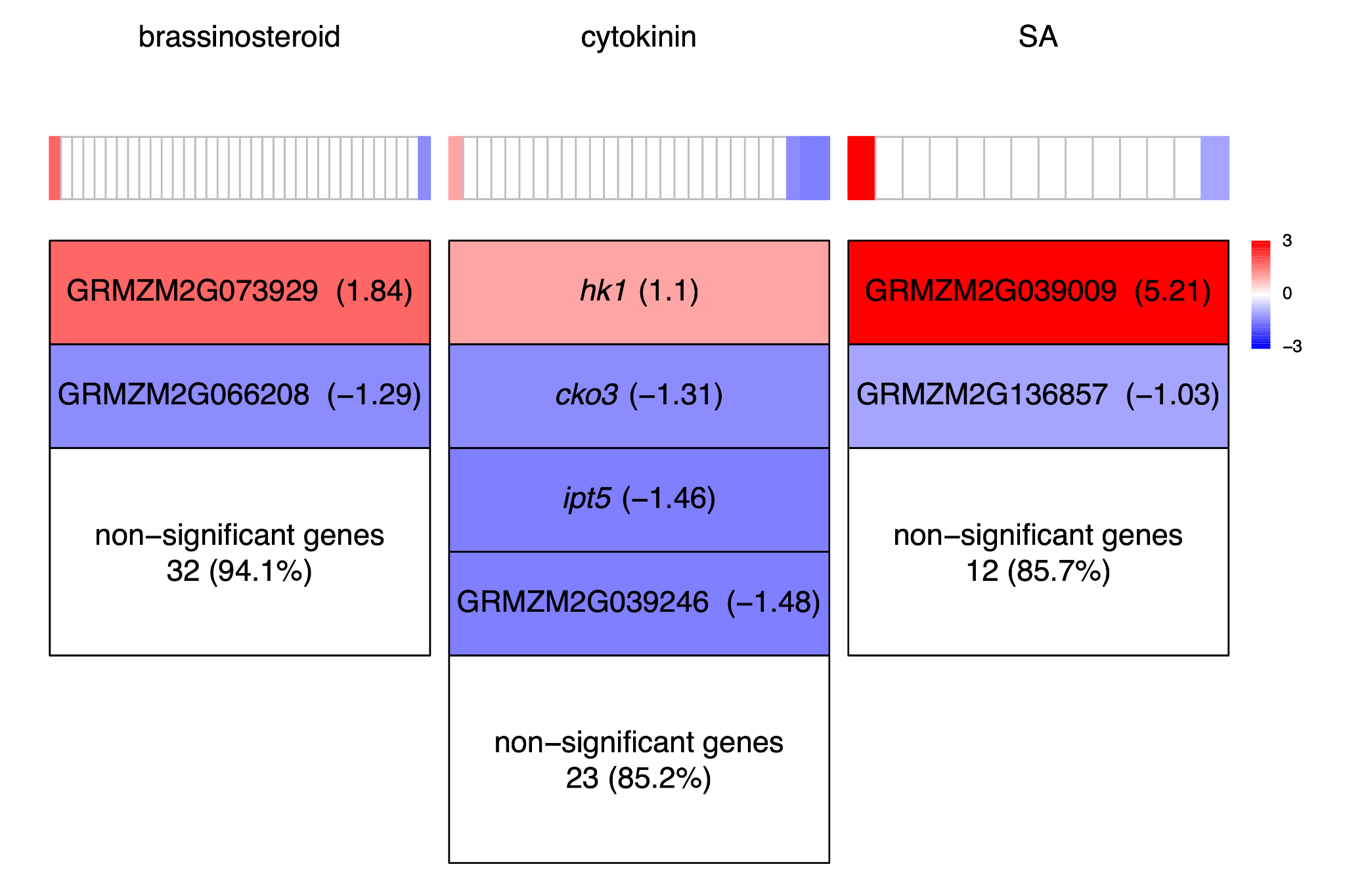


**Figure S5**. List of significant DE genes in three hormone pathways. The log2 Cn:mock fold change of gene expression (log2FC) averaged from both R and S lines are listed after each gene name. Only genes with adjusted p-values less than 0.05 and absolute log2FC larger than 1 are displayed.


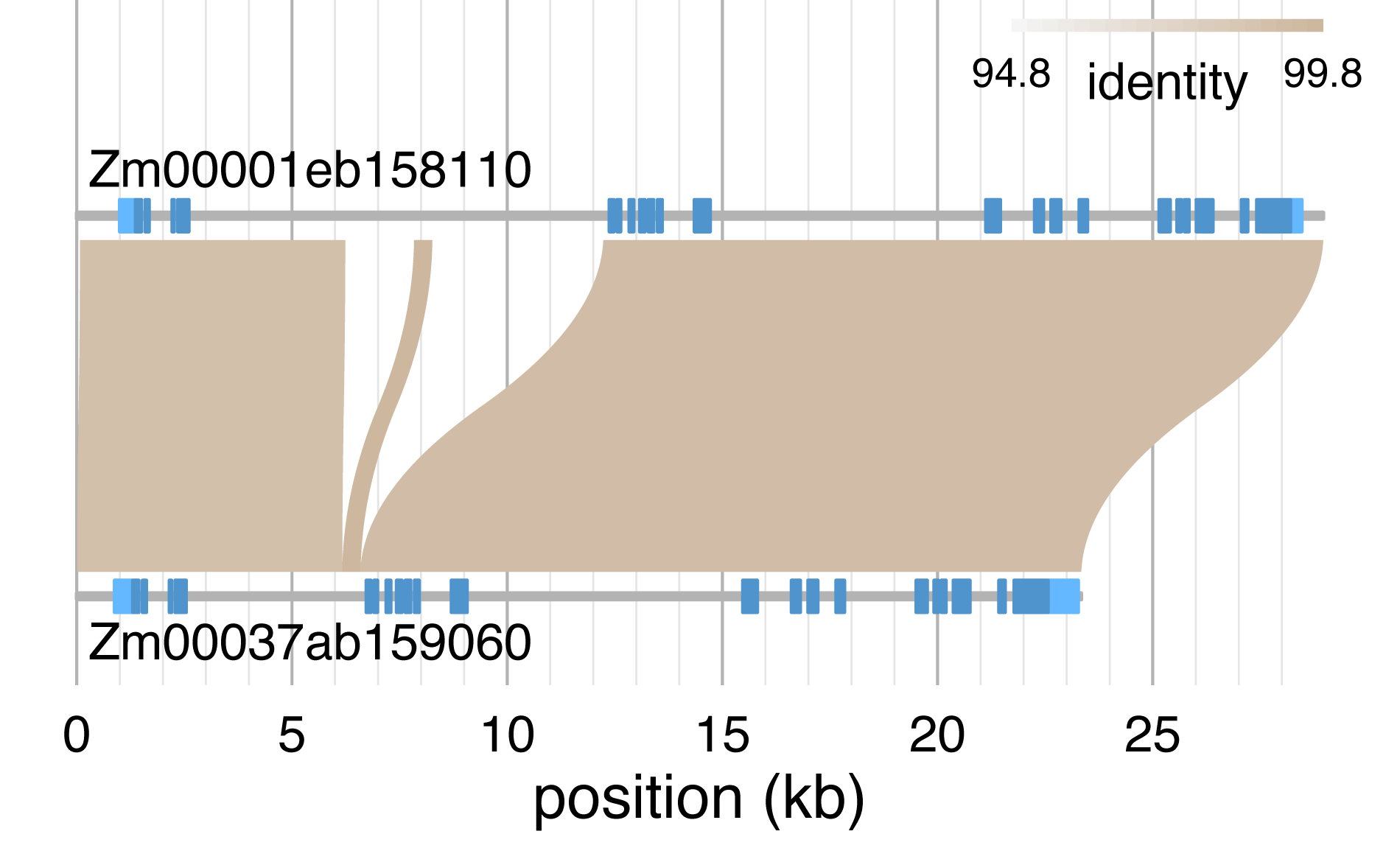


**Figure S6.** Sequence alignments between candidate gene Zm00001eb158110 from B73 and Zm00037ab159060 from NC358. Dark and light blue colors represent coding and untranslated regions, respectively.


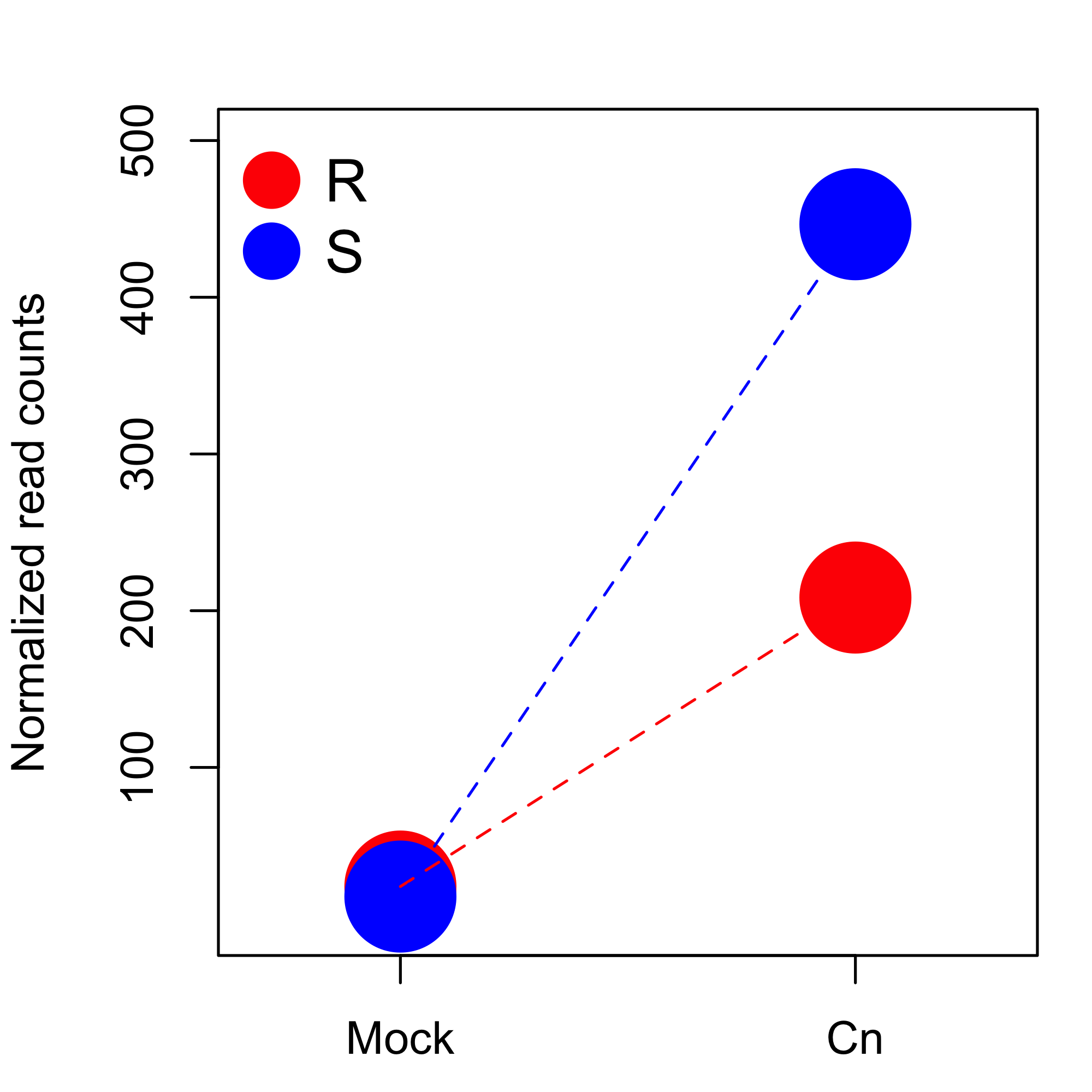


**Figure S7**. Expression of GRMZM2G033515. Normalized read counts of GRMZM2G033515 in R and S pools in the control (Mock inoculation) and in the Cn treatment are plotted.
